# Role of vascular channels as a novel mechanism for subchondral bone damage at cruciate ligament entheses in osteoarthritis and inflammatory arthritis

**DOI:** 10.1136/annrheumdis-2013-203972

**Published:** 2013-10-04

**Authors:** D A Binks, E M Gravallese, D Bergin, R J Hodgson, A L Tan, M M Matzelle, D McGonagle, A Radjenovic

**Affiliations:** 1 Leeds Institute of Rheumatic and Musculoskeletal Medicine, University of Leeds, Leeds, UK; 2 Leeds Musculoskeletal Biomedical Research Unit, National Institute for Health Research, Chapel Allerton Hospital, University of Leeds, Leeds, UK; 3 Department of Medicine, University of Massachusetts Medical School, Worcester, Massachusetts, USA; 4 Department of Radiology, Galway University Hospitals, Galway, Ireland

**Keywords:** Osteoarthritis, Inflammation, Magnetic Resonance Imaging, Rheumatoid Arthritis, Knee Osteoarthritis

## Abstract

**Objectives:**

The purpose of this work was to test whether normal peri-entheseal vascular anatomy at anterior and posterior cruciate ligaments (ACL and PCL) was associated with distribution of peri-entheseal bone erosion/bone marrow lesions (BMLs) in inflammatory arthritis (IA) and osteoarthritis (OA).

**Methods:**

Normal microanatomy was defined histologically in mice and by 3 T MRI and histology in 21 cadaveric knees. MRI of 89 patients from the Osteoarthritis Initiative and 27 patients with IA was evaluated for BMLs at ACL and PCL entheses. Antigen-induced arthritis (AIA) in mice was evaluated to ascertain whether putative peri-entheseal vascular regions influenced osteitis and bone erosion.

**Results:**

Vascular channels penetrating cortical bone were identified in knees of non-arthritic mice adjacent to the cruciate ligaments. On MRI of normal cadavers, vascular channels adjacent to the ACL (64% of cases) and PCL (71%) entheses were observed. Histology of 10 macroscopically normal cadaveric specimens confirmed the location of vascular channels and associated subclinical changes including subchondral bone damage (80% of cases) and micro-cyst formation (50%). In the AIA model, vascular channels clearly provided a site for inflammatory tissue entry and osteoclast activation. MRI showed BMLs in the same topographic locations in both patients with early OA (41% ACL, 59% PCL) and IA (44%, 33%).

**Conclusion:**

The findings show that normal ACL and PCL entheses have immediately adjacent vascular channels which are common sites of subtle bone marrow pathology in non-arthritic joints. These channels appear to be key determinants in bone damage in inflammatory and degenerative arthritis.

## Introduction

A prominent feature of rheumatoid arthritis (RA) is periarticular joint destruction that is associated with poor functional outcome.^1^ Synovitis is the primary lesion in RA and is followed by osteitis, osteoclastic mediated joint destruction and the later appearance of characteristic erosions.^2^ Somewhat surprisingly, normal joints exhibit microscopic erosions on high resolution CT, MRI and on histological assessment at the same topographical locations.^3^ We and others have noted that erosions in the small joints at typical peri-ligamentous and entheseal locations likely reflect a common biomechanical drive.^3–5^ Indeed, such erosions occur in an identical topographic location in hand osteoarthritis (OA), pointing towards common anatomical factors in joint damage, irrespective of the primary pathology.^6^

Large joints, including the knee, are also associated with erosion formation and periarticular damage in RA, spondyloarthritis (SpA) and sometimes OA. Although ‘bone oedema’, or what are known as bone marrow lesions (BMLs) in OA, has been linked to sites of articular cartilage denudation,^7^ ^8^ BMLs are also commonly seen at the anterior cruciate ligament (ACL) insertion, immediately proximal to it, or indeed within the underlying trabecular bone.^9^ These enthesis-related bone lesions in OA are reminiscent of the small joint enthesis-centred bone pathology that is evident in the hand in early generalised OA and RA.^3^

Unlike in the small joints, the microanatomical basis for large joint bone damage including BMLs and erosion formation has not been well studied. Anecdotally, we noted that bone damage/erosion in experimental arthritis occurs where blood vessels enter the bone. Consequently, we hypothesised that the hitherto poorly defined vascular anatomy of large human entheses may play a role in bone damage in inflammatory and degenerative arthritis. In this work, we report on a novel mechanism that appears to contribute to joint damage in inflammatory and degenerative arthritis in man and in the experimental setting.

## Methods

### Antigen-induced arthritis

All animal work was approved by the animal ethics committee at University of Massachusetts Medical School. C57BL/6 mice were purchased from Jackson Laboratories and studied at 12 weeks of age. To induce arthritis, methylated bovine serum albumin (BSA) (4 mg/mL; Sigma) was emulsified in an equal volume of Freund's complete adjuvant (CFA, Sigma) containing 2.5 mg/mL heat-killed *Mycobacterium tuberculosis* (strain H37Ra; Difco, Detroit, Michigan, USA). At day -7, mice were immunised by subcutaneous injection of 100 µL mBSA/CFA emulsion into the base of the tail. On day 0, arthritis was induced by injecting mice intra-articularly with 10 µg/µL of mBSA in phosphate buffered saline (PBS) in a 6 µL volume into the left knee; the right knee received a 6 µL injection of PBS as a control. On day 0, mice were also boosted with 200 µg/mL lipopolysaccharide (LPS) (Sigma) in a 100 µL volume via intraperitoneal injection.

Hind limbs were fixed in 4% paraformaldehyde (PFA) for 24 h, decalcified in 15% EDTA/0.5% PFA, and paraffin embedded. For histologic evaluation of inflammation and bone erosion, sections were stained with either H&E or tartrate-resistant acid phosphatase (TRAP). For TRAP staining, sections were incubated in 0.1 M Tris-HCl, pH 9.0 for 18 h, followed by 0.1 M sodium citrate, pH 5.2 for 3 h.^10^ Activated sections were incubated for 30 min at 37°C in 0.005% Napthol AS-MX phosphate (Sigma)/0.01% N,N-dimethylformamide/0.03% Fast Red Violet LB salt (Sigma)/50 mM sodium tartrate in 0.1 M acetate pH 5.0 11 12 counterstained with haematoxylin.

### MRI of normal cadaveric knees

**Figure 1:**
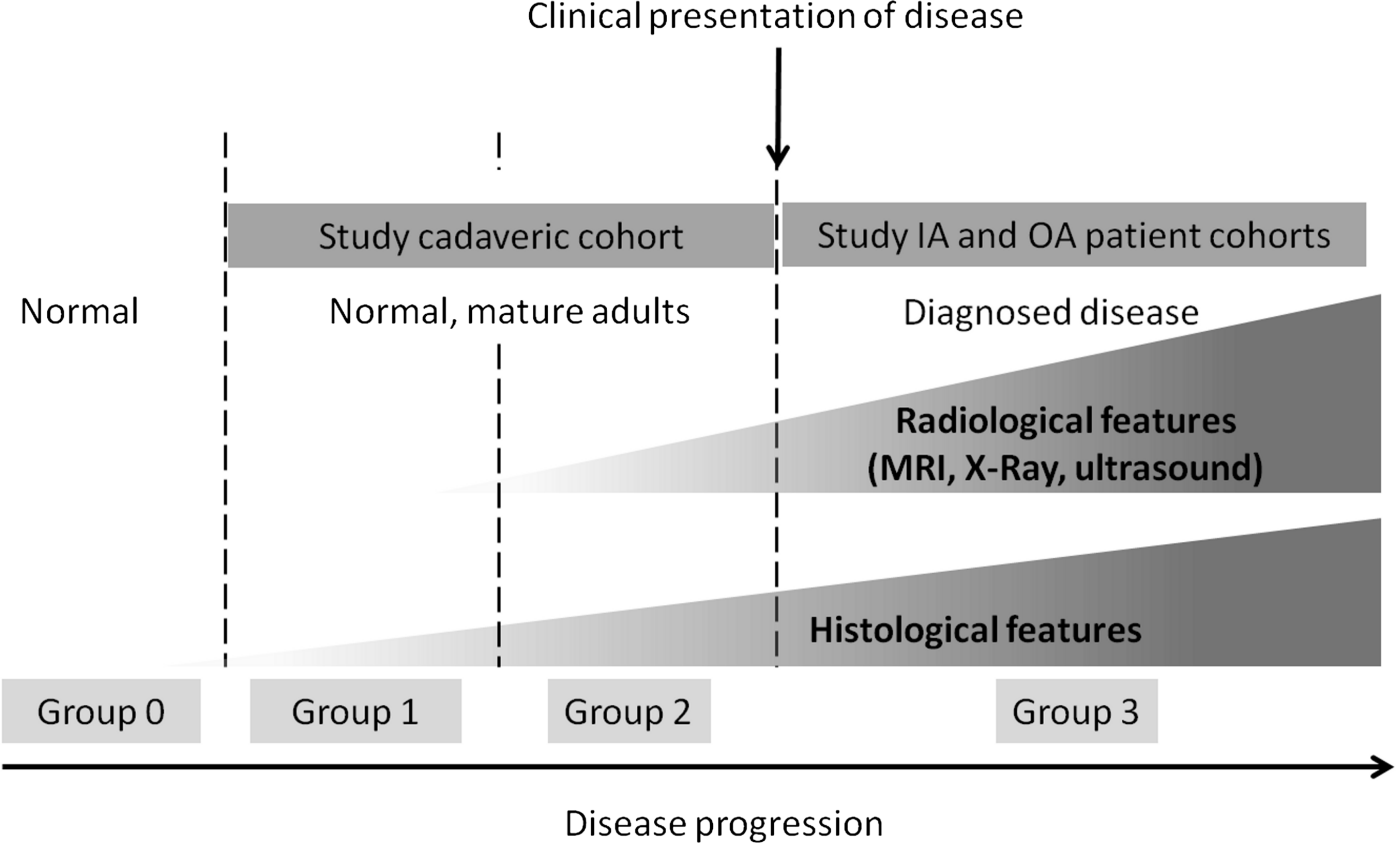
Progression of histological and radiological features of disease relative to clinical presentation. Completely normal tissue is defined as that which lacks histological and radiological features (group 0). This likely corresponds to young healthy people of which none were included in this study. Group 1 represents normal subjects without clinical arthritis but in which some microscopic histological subclinical age related degenerative features were present. Group 2 represents more severe forms of subclinical disease in which radiological features were seen in addition to the histological features present in group 1. Group 3 corresponds to clinically evident arthritis and is represented by the IA and osteoarthritis patient cohorts that underwent MRI in the present study. Cadaveric specimens were not collected from donors who had a documented history of arthritis. The work in this study focussed on groups 1 and 2 (preclinical imaging and histological disease) and group 3 (clinically demonstrable disease). Accordingly there may be some overlap between these groups.

Whole human knee joints were obtained from the Leeds Tissue Bank^13^ for the purpose of obtaining high-resolution 3 T MRI images and comparative histopathology. The study was approved by the Local Research Ethics Committee (LREC) and all donors gave informed consent. Samples were collected from donors, none of whom had an ante-mortem history of knee arthritis. No samples were obtained from young donors, with cadaveric cohort specimens being obtained from normal mature adults in the age group where degenerative arthritis is typically seen (figure 1). Whole joint MRI was performed on 14 cadaveric knees (6 male, 8 female, mean age=69.9). Histopathological analysis was performed on 10 specimens (5 male, 5 female, mean age=63.9), including 3 samples which had also undergone the whole joint MRI protocol. MRI images were acquired on a 3.0 T Siemens Verio system using an 8-channel knee coil. The examination protocol was based on the National Institutes for Health (NIH) Osteoarthritis Initiative (OAI) MRI knee protocol.^14^ This includes *T*_1_-, *T*_2_^*^- and intermediate-weighted sequences with sagittal and coronal acquisitions in 2-D and 3-D. In addition to the basic OAI protocol, selected protocols were repeated with increased in-plane and slice resolution to afford high-resolution images with the expense of increased acquisition times.

The cadaveric MRI images were scored by a musculoskeletal radiologist (RJH) for the unequivocal presence of the following features: ACL and posterior cruciate ligament (PCL) full thickness tears; loss of continuous low signal line at ACL and PCL insertions taken as corresponding to cortical bone disruption; presence of a thin high signal line posterior to ACL insertion taken as corresponding to tibial vascular channel; presence of a thin high signal line anterior to PCL insertion take as corresponding to tibial vascular channel; focal high signal intensity within 1 cm of the bone surface corresponding to BMLs. Four regions were assessed for the presence of BMLs: (i) the region immediately adjacent to the ACL insertion, (ii) immediately posterior to the ACL insertion, (iii) immediately anterior to the PCL insertion and (iv) immediately adjacent to the PCL insertion.

### Histopathology of cadaveric specimens

Histological specimens were obtained by removing the mediolateral central portion of the tibial plateau containing the cruciate ligament insertions. The dissected tissue blocks were fixed in formalin prior to decalcification in 5% formic acid and embedded in paraffin wax. Sagittal sections 4 microns thick were cut at 1 mm intervals across the tissue block. Adjacent sections were stained with H&E and Masson's trichrome. We evaluated the vascular microanatomy at the ACL and PCL tibial entheses and recorded the presence of bone damage and degenerative changes in these regions including trabecular destruction, cystic changes and accumulation of myxoid material.

### MRI of patients with early OA 

Data used in the preparation of this article were obtained from the OAI database, which is available for public access at http://www.oai.ucsf.edu/.^15^ Two cohorts from the OAI were evaluated separately for the presence of ACL and PCL lesions, respectively, by one musculoskeletal radiologist (DB). PCL lesions were evaluated from 3 T MRI images of 98 knees representing 49 patients (mean age=59.8) from the progression cohort of the OAI study. The specific dataset used was Image Release 0.B.2. ACL lesions were evaluated using MRI images of 80 knees representing 40 patients (mean age=62.2) selected from the OAI Image Release V.0.A.2, including patients from the Progression and Incidence sub-cohorts. In both cases, sagittal and coronal fluid sensitive MRI sequences were used to evaluate the presence of tibial vascular channels at the ACL or PCL insertions. The presence of BMLs was noted and graded for severity (1–3) in the same four regions defined above. The integrity of the ACL and PCL fibres was assessed and graded from 0 (normal) to 3 (full thickness PCL tear). Disruption of the cortical bone at the ACL and PCL attachment was recorded as present or not and the dimensions of intraosseous cysts at the attachments were recorded.

### MRI of patients with IA 

Twenty seven patients (mean age=37.8, 18 male, 9 female) with IA (14 cases of psoriatic arthritis (PsA), six undifferentiated arthritis, four RA and three reactive arthritis (ReA)) were recruited to the study. The mean disease duration was 15 months; mean erythrocyte sedimentation rate was 25 mm/h; mean C-reactive protein was 29 mg/L. Patients were recruited from the rheumatology outpatient clinics in the Leeds Teaching Hospital Trust. All patients had IA and presented with a swollen knee. The study was approved by the LREC and written informed consent was obtained from all patients. The knee was scanned using a Phillips Gyroscan ACS NT 1.5 T scanner. T_1_-weighted, T_2_-weighted selective partial inversion recovery (T_2_-SPIR), and pre and post contrast T_1_-weighted SPIR sequences were performed. The presence of BMLs was noted and graded for severity (1–3) in the same four compartments outlined above. Images were scored in consensus by two readers (DMcG and ALT).

## Results

### Animal model data

**Figure 2:**
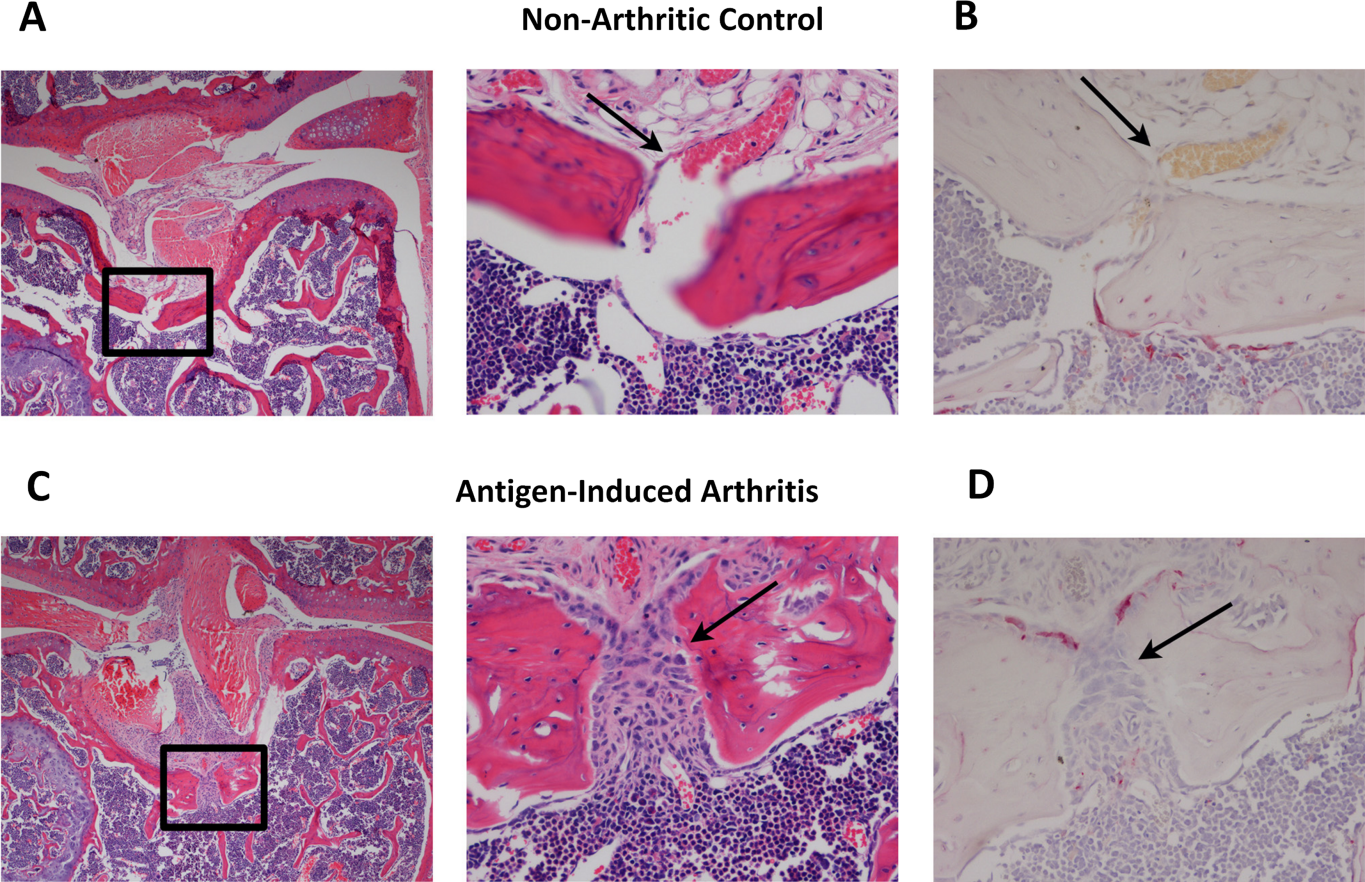
(A) Non-Arthritic: Knee joint of control, non-arthritic mouse. Boxed region (left) is enlarged (right) and demonstrates a large blood vessel penetrating through bone and into the marrow space in the region of anterior cruciate ligament/posterior cruciate ligament attachment. (B) tartrate-resistant acid phosphatase (TRAP) stain shows TRAP activity at this site. (C) Arthritic: Boxed region (left) shows similar anatomic region to that shown in A and B and is enlarged (right). Inflammatory tissue enters the bone marrow space via blood vessel channel. (D) TRAP stain shows TRAP expressing cells at interface between inflammation and bone.

Evaluation of samples from non-arthritic mice revealed the presence of vascular channels at sites of cruciate ligament attachment in the knee joint. These channels penetrated through the bone cortex and into the marrow space (figure 2A). Interestingly, TRAP activity was often seen at sites of vessel penetration, suggesting that this may be a site of bone remodelling (figure 2B). Induction of antigen-induced arthritis (AIA) resulted in significant inflammation in the knee joint. We noted that these sites of vessel penetration through the bone cortex were common entry points of synovially-derived inflammatory tissues into the marrow space (figure 2C). Furthermore, TRAP-expressing multinucleated cells were commonly seen at these sites, accompanied by bone erosion and expansion of channel width (figure 2D). These murine studies showed a clear anatomical contribution in erosion formation and prompted a close evaluation of human knee joints, to determine whether similar structures existed.

### MRI of normal cadavers

**Figure 3:**
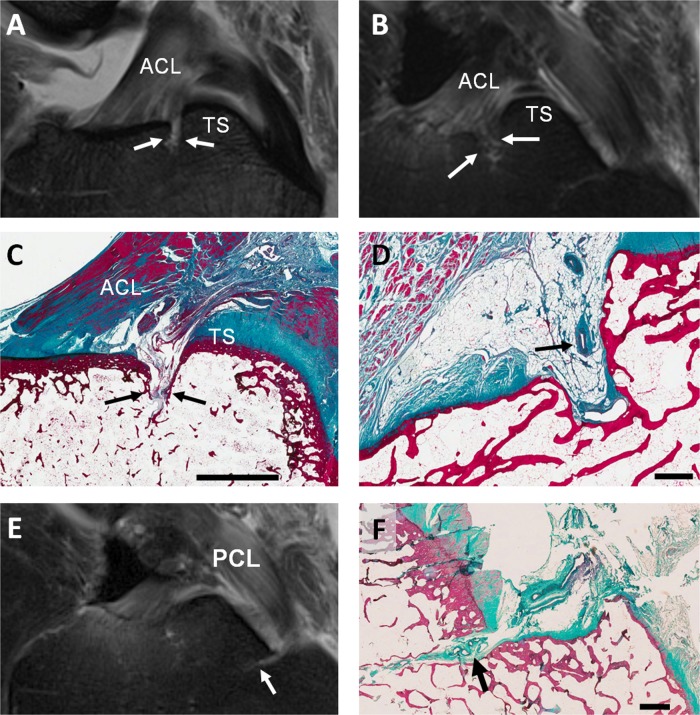
Vascular channels posterior to the site of the anterior cruciate ligament (ACL) tibial insertion: (A–B) sagittal intermediate-weighted turbo spin-echo MR images of normal cadaveric tissue samples. (A) The vascular channel is seen as a thin high signal line penetrating the bone cortex (arrows) anterior to the tibial spines (TS). (B) Bone marrow oedema observed at the expected location of the vascular channel (arrows). (C) Corresponding sagittal histological section of the same cadaveric sample shown in A, showing a vascular channel (arrows) penetrating the bone cortex into the marrow space. These vascular channels are conduits for vascular penetration through the bone cortex. (D) Sagittal histological section showing blood vessels (arrow) in a vascular channel posterior to the ACL insertion. Vascular channel immediately anterior to the tibial insertion of the posterior cruciate ligament (PCL): (E) Sagittal fluid-sensitive MRI image showing high signal region corresponding to vascular channel (arrow) in age normal cadaveric tissue. (F) Corresponding sagittal histological section of the same cadaveric sample in E, shows a vascularised channel (arrow) with its origin immediately anterior to the tibial insertion of the PCL, extending anteriorly into the subchondral bone. Scale bars; C=5 mm, D and F=1 mm.

On 3 T MRI images of normal cadaveric tissue the vascular channel posterior to the ACL insertion appeared as a region of hyperintensity on intermediate-weighted fat suppressed sequences (figure 3A). This region of hyperintensity was identified in 64% of the 14 cadaveric MRI datasets reviewed. In certain cases it was not possible to distinguish unequivocally the presence of a vascular channel, particularly where oedematous features appeared at the sites overlapping with the expected location of the channels (figure 3B); in the five cases where a vascular channel was not identified, focal high signal corresponding to bone marrow oedema was present in four. In all, high signal attributed to a vascular channel or to bone marrow oedema in this region was identified in 93% of knees examined. There was no incidence of ACL tear in the 14 samples examined. Disruption of the cortical bone at the ACL insertion was apparent in 50% of cases. BMLs adjacent to the ACL insertion were present in 71%. BMLs in the region immediately posterior to the ACL insertion (ie, the region containing the vascular channel) were present in 71% of cases.

At the PCL insertion, a vascular channel was identified in 71% of cases (figure 3E). Again, in some cases it was not always possible to distinguish between high signal corresponding to presence of a vascular channel or bone marrow oedema. A vascular channel was clearly visualised in 10 out of 14 cases and bone marrow oedema in the region where we would expect to observe the vascular channel was seen in the remaining four cases. There was no incidence of PCL tear. Disruption of the cortical bone at the PCL insertion was seen in 7% of cases. BMLs adjacent to the PCL insertion were present in 50% of knees and BMLs immediately anterior to the PCL insertion were seen in 50% of knees.

### Histopathology of normal cadavers

**Figure 4:**
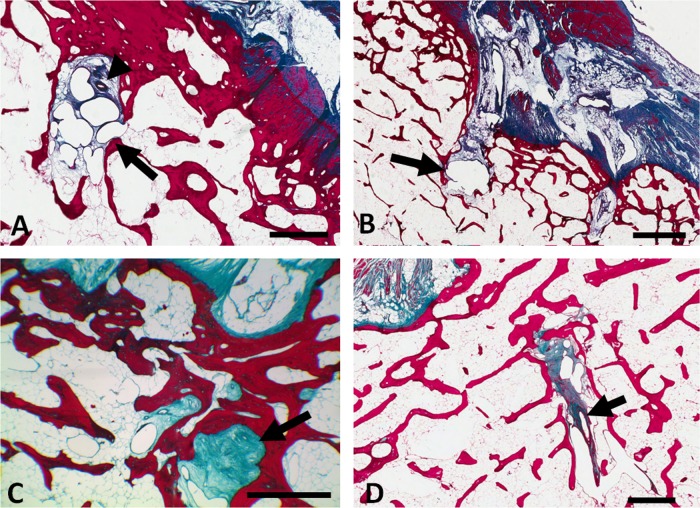
Microanatomical damage associated with the anterior cruciate ligament (ACL) vascular channel. Sagittal histological sections showing: (A) Accumulation of micro-cysts (arrow) in the ACL vascular channel and blood vessel directly above (arrowhead). (B) Accumulation of fluid (arrow) at the terminus of the ACL vascular channel; (C) Collagenous/myxoid material (arrow) present in the vascular channel; (D) Subchondral extension of the vascular channel and presence of blood vessel (arrow). Scale bars; A, C and D=1 mm, B=2 mm.

We histologically examined the region immediately posterior to the tibial attachment of the ACL in 10 normal cadaveric specimens. A vascular channel posterior to the ACL insertion and anterior to the tibial spines (figure 3C) was clearly evident in all but one specimen surveyed (in the remaining sample, the region of interest was obscured by tissue preparation artefacts). In all cases where the channel could be seen, micro-vessels were also present in the channel (figure 3D). In addition to the almost universal presence of a vascularised channel at the ACL insertion, we also noted a continuum of associated microanatomical damage of varying severity (figure 4A–D). In 80% of the cases studied we saw subchondral bone damage whereby the vascular channel was seen to extend deep into the subchondral bone and also into adjacent sections. Cystic formation/fluid accumulation (50%) was also observed in conjunction with this subchondral bone damage. In 20% of subjects examined, accumulation of myxoid^16^ material within the vascular channel was observed. Typically these features were associated with the termini of the vascular channels and were less prevalent at the cortical origin. We also saw a vascular channel and associated microvasculature immediately anterior to the tibial insertion of the PCL, though less frequently (40%) than at the ACL (figure 3F). Again, we noted the presence of degenerative histological changes including cyst formation (10%) and myxoid material (30%) associated with the vascular channel as seen at the ACL insertion.

### Patient MRI

**Figure 5:**
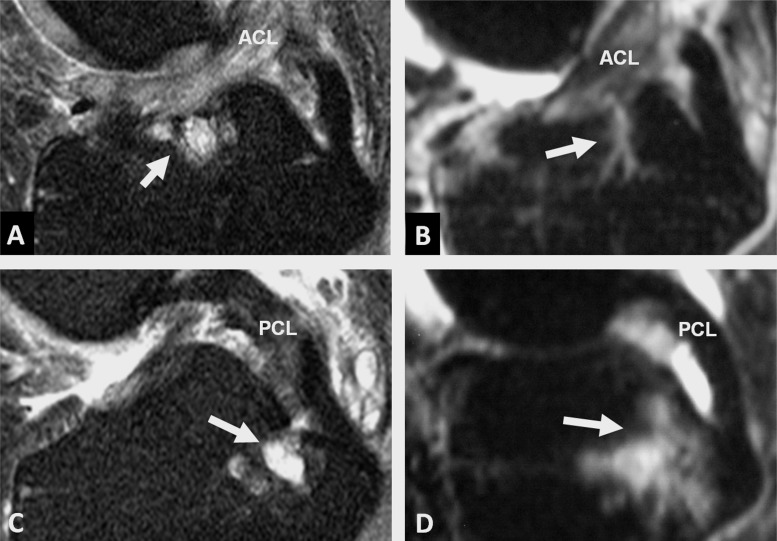
Sagittal fluid-sensitive MRI images showing oedematous features (arrows) adjacent to the vascular channels in patients with osteoarthritis (OA) and inflammatory arthritis. Bone marrow lesions adjacent to the anterior cruciate ligament (ACL) vascular channel in A, patient with early OA and B, patient with undifferentiated arthritis. Bone marrow lesions and intraosseous cysts adjacent to the posterior cruciate ligament (PCL) vascular channel in C, patient with early OA and D, patient with psoriatic arthritis.

In the patient cases scored from the OAI cohort, the presence of the ACL tibial vascular channel was recorded in 94% of cases. The ACL was graded as normal with respect to tearing in 93% of knees; grade 1, corresponding to localised high signal, in 3%; and grade 3, corresponding to full thickness ACL tear, in 4%. BMLs adjacent to the site of the tibial ACL insertion were observed in 63% of knees studied. BMLs immediately posterior to the site of insertion were observed in 41% of cases (figure 5A). Disruption of the cortical bone at the ACL insertion was seen in 76% of knees. One or more intraosseous cysts were observed in 23% of knees. At the PCL, a vascular channel was observed in 70% of knees. The PCL was graded as normal with respect to tearing in 77% of knees; grade 1, corresponding to localised high signal, in 14%; and grade 2, corresponding to a partial thickness tear, in 8%. No incidence of full thickness PCL tear was observed. BMLs adjacent to the PCL insertion were seen in 52% of cases and BMLs immediately anterior to the PCL insertion were seen in 59% (figure 5C). Disruption of the bone cortex at the PCL insertion was seen in 21% of cases. Intraosseous cysts at the PCL insertion were seen in 42% of knees.

### Bone marrow lesions in IA

In the IA patient cohort, we noted BMLs adjacent to the ACL insertion in 26% of cases. BMLs immediately posterior to the ACL insertion were present in 44% of patients (figure 5B). BMLs were recorded in 26% of cases in the region immediately adjacent to the PCL insertion and in 33% of cases in the region immediately anterior to the PCL tibial insertion (figure 5D).

A summary of the histological and MRI features assessed in each of the patient cohorts and the cadaveric cohort is presented in table 1.

**Table 1:** Frequency of MRI features observed in normal cadaveric specimens, a cohort of patients with early osteoarthritis and a cohort of patients with IA

	Cadaveric cohort (%)	Early OA cohort (%)	IA cohort (%)
MRI features			
ACL vascular channel	64	94	–
Oedema in overlapping region	29	–	–
Total	93	94	–
Bone marrow lesions at ACL insertion	71	63	26
Bone marrow lesions immediately posterior to ACL insertion	71	41	44
ACL tear	0	7	–
Cortical bone disruption at ACL insertion	50	76	–
Intraosseous cysts at ACL insertion	–	23	–
PCL vascular channel	71	70	–
Oedema in overlapping region	29	–	–
Total	100	–	–
Bone marrow lesions at PCL insertion	50	52	26
Bone marrow lesions immediately anterior to PCL insertion	50	59	33
PCL tear	0	23	–
Cortical bone disruption at PCL insertion	7	21	–
Intraosseous cysts at PCL insertion	–	42	–
Histology features			
ACL vascular channel	90	–	–
ACL channel sub chondral bone changes	80	–	–
ACL channel microcysts	50	–	–
ACL channel myxoid accumulation	20	–	–
PCL vascular channel	40	–	–
PCL channel microcysts	10	–	–
PCL channel myxoid accumulation	30	–	

The frequency of histologically observed features is also presented for the cadaveric cohort.

ACL, anterior cruciate ligament; IA, inflammatory arthritis; OA, osteoarthritis; PCL, posterior cruciate ligament.

## Discussion

The purpose of this work was to explore the role of bone peri-entheseal penetrating vascular channels as possible drivers in the pathogenesis of joint damage in inflammatory and degenerative arthritis in human and experimental model systems. Here, we demonstrated a continuum of features in the murine model ranging from knee joint peri-entheseal vascular channels, TRAP expressing cells in normal non-inflamed joints, to frank erosion in IA at the same location. In humans, we noted identical channels with associated damage that ranged from degenerative histological changes and micro-erosion in non-arthritic joints to BMLs in the same topographic location in established inflammatory and degenerative disease. This anatomical configuration of normal enthesis vascularity appears to be a key determinant of joint damage in inflammatory and degenerative arthritis.

Importantly, the pathologic origin of bone marrow lesions observed using MRI differs between OA and IA. In IA, Jimenez-Boj et al have shown that MRI signal changes observed in the metacarpophalangeal (MCP) joints of patients with RA corresponded histologically to infiltration of inflammatory material,^17^ whereas the histological correlates of bone marrow lesions in OA include fibrosis, necrosis, trabecular remodelling and marrow bleeding but not inflammation.^18^ Hernández-Molina et al have previously reported on central BMLs abutting the ACL in a cohort of patients with symptomatic OA and suggested an entheseal role in their aetiology.^9^ Our observations may provide a link between peri-entheseal vascular channels and the observed pattern of central BMLs and the potential for subsequent ACL failure. While a limitation of this study is that we only report on a relatively small cross section of patients suffering from arthritis, the frequency with which we observed lesions adjacent to vascular channels in OA and IA shows the relevance of this site to the underlying disease process. We propose that these vascular channels are sites of stress and degenerative change in normals but in disease this is associated with more extensive lesions. This has important implications for understanding the mechanisms of tissue damage in OA and in IA. In support of this, we noted that in experimental models of severe joint inflammation a florid inflammatory reaction could be seen in bone at this site of relative weakness. What is the mechanism for erosion formation in this setting? Firstly, the pulsality of blood vessels will ensure that the space between the vessel and the bone is quite literally not ‘watertight’. Consequent with the elevation in the intra-articular pressure that might take place during inflammation, it is possible that fluid and inflammatory mediated cells are forced down this channel into the adjacent bone, particularly at sites of high mechanical activity. The homeostatic tissue repair associated with such a scenario likely explains the findings in normal cadaveric joints. The variable inflammatory milieu associated with clinical arthritis likely exaggerates this response and it manifests in disease.

Thus far, specific consideration to the role of such lesions in pain and progressive joint destruction has not been evaluated. It is possible that damage underneath the ACL insertion in particular could contribute to ACL failure in OA. The specific role of bone oedema at this site in joint pain is something that also merits consideration. Clearly, strategies aimed at cartilage repair are likely to be of limited value in these ligamentogenic-entheseogenic patterns of OA.

Previous experimental work has shown early osteoclastic activation in murine AIA in the periosteum-synovium-cartilage junction and within the Haversian channels, with these locations seemingly providing entry points for synovitis and subsequent osteoclast-mediated joint destruction.^19^ Marinova-Mutafchieva *et al* have reported on vascularised bone canals and their putative role in the migration of mesenchymal stem cells in a murine collagen-induced arthritis model, but no actual migration was shown.^20^ To the best of our knowledge the present findings represent a novel mechanism contributing to joint damage.

In conclusion we have described a novel mechanism for microdamage in normal joints and a mechanism contributing to bone damage in inflammatory and degenerative arthritis. There appears to be a continuum of damage from normal vascular channels adjacent to the ACL/PCL through to intermediate microanatomical damage and finally established erosions at the same location. To what degree lesions at these sites contribute to joint damage and how this may be modified opens up new avenues for understanding joint damage.
